# Facial Expression Recognition Based on Local Binary Patterns and Kernel Discriminant Isomap

**DOI:** 10.3390/s111009573

**Published:** 2011-10-11

**Authors:** Xiaoming Zhao, Shiqing Zhang

**Affiliations:** 1 Department of Computer Science, Taizhou University, Taizhou 317000, China; 2 School of Physics and Electronic Engineering, Taizhou University, Taizhou 318000, China; E-Mail: tzczsq@163.com

**Keywords:** kernel, isometric mapping, dimensionality reduction, local binary patterns, facial expression recognition

## Abstract

Facial expression recognition is an interesting and challenging subject. Considering the nonlinear manifold structure of facial images, a new kernel-based manifold learning method, called kernel discriminant isometric mapping (KDIsomap), is proposed. KDIsomap aims to nonlinearly extract the discriminant information by maximizing the interclass scatter while minimizing the intraclass scatter in a reproducing kernel Hilbert space. KDIsomap is used to perform nonlinear dimensionality reduction on the extracted local binary patterns (LBP) facial features, and produce low-dimensional discrimimant embedded data representations with striking performance improvement on facial expression recognition tasks. The nearest neighbor classifier with the Euclidean metric is used for facial expression classification. Facial expression recognition experiments are performed on two popular facial expression databases, *i.e*., the JAFFE database and the Cohn-Kanade database. Experimental results indicate that KDIsomap obtains the best accuracy of 81.59% on the JAFFE database, and 94.88% on the Cohn-Kanade database. KDIsomap outperforms the other used methods such as principal component analysis (PCA), linear discriminant analysis (LDA), kernel principal component analysis (KPCA), kernel linear discriminant analysis (KLDA) as well as kernel isometric mapping (KIsomap).

## 1. Introduction

Facial expressions are the facial changes indicating a person’s internal affective states, intentions or social communications. Based on the shown facial expressions, human face is the predominant mode of expressing and interpreting affective states of human beings. Automatic facial expression recognition impacts important applications in many areas such as natural human computer interactions, image retrieval, talking heads and human emotion analysis [1]. Over the last decade, automatic facial expression recognition has been increasingly attracting attention and has become an important issue in the scientific community, since facial expressions are one of the most powerful, nature and immediate means for human beings to communicate their emotions and intentions.

A basic automatic facial expression recognition system generally consists of three steps [2]: face acquisition, facial feature extraction and representation, and facial expression classification. Face acquisition is a preprocessing stage to detect or locate the face region in input images or sequences. The real-time face detection algorithm developed by Viola and Jones [3] is the most commonly employed face detector, in which a cascade of classifiers is employed with Harr-wavelet features. Based on the eye position detected in the face region, the detected face region is usually aligned. After detecting or locating the face, the next step is to extract facial features from original face images to represent facial expressions. There are mainly two approaches to this task: geometric features-based methods and appearance features-based methods [2]. Geometric features present the shape and locations of facial components such as mouth, nose, eyes, and brows. Nevertheless, the geometric feature extraction requires accurate and reliable facial feature detection, which is difficult to realize in real time applications. More crucially, geometric features usually cannot encode changes in skin texture such as wrinkles and furrows that are critical for facial expression modeling. In contrast, appearance features present appearance changes (skin texture) of the face, including wrinkles, bulges and furrows. Image filters, such as principal component analysis (PCA) [4], linear discriminant analysis (LDA) [5], Gabor wavelet analysis [6–9], can be applied to either the whole-face or specific face regions to extract facial appearance changes. However, it is computationally expensive to convolve face images with a set of Gabor filters to extract multi-scale and multi-orientation coefficients. It is thus inefficient in both time and memory for high redundancy of Gabor wavelet features [10–13]. In recent years, local binary patterns (LBP) [10], originally proposed for texture analysis and a non-parametric method efficiently summarizing the local structures of an image, have received increasing interest for facial expression representation. The most important property of LBP features is their tolerance against illumination changes and their computational simplicity. LBP has been successfully applied as a local feature extraction method in facial expression recognition [11–13]. The last step of an automatic facial expression recognition system is to classify different expressions based on the extracted facial features. A variety of classifiers, such as Neural Networks (NN) [14], Support Vector Machines (SVM) [15], k-Nearest Neighbor (kNN) [16], rule-based classifiers [17], and Hidden Markov Models (HMM) [18], have been used for facial expression recognition.

In the second step of an automatic facial expression recognition system, the extracted facial features are represented by a set of high-dimensional data. Therefore, it would be desired to analyze facial expressions in the low-dimensional subspace rather than the ambient space. To solve this problem, there are mainly two methods: linear and nonlinear. The well-known linear methods are PCA and LDA. However, they are not suitable for representing dynamically changing facial expressions, which can be represented as low-dimensional nonlinear manifolds embedded in a high-dimensional image space [19,20]. Considering the nonlinear manifold structure of facial images, manifold learning (also called nonlinear dimensionality reduction) methods, which aim to find a smooth low-dimensional manifold embedded in a high-dimensional data space, have been recently applied to facial images for automatic facial expression analysis [21,22]. The two representative manifold learning methods are locally linear embedding (LLE) [19] and isometric feature mapping (Isomap) [20]. By using manifold learning methods such as LLE and Isomap, facial expression images can be projected to a low-dimensional subspace in which facial data representation is optimal for classification. However, these methods lack a good generalization property on new data points since they are defined only on training data.

To overcome the above drawback of manifold leaning methods, some kernel-based manifold learning methods, like kernel isometric mapping (KIsomap) [23], have been recently developed. KIsomap effectively combines the kernel idea and Isomap and can directly project new data points into a low-dimensional space by using a kernel trick as in kernel principal component analysis (KPCA) [24]. However, this kind of KIsomap algorithm still has two shortcomings. First, KIsomap fails to extract the discriminant embedded data representations since KIsomap does not take into account the known class label information of input data. Second, as a kernel-based method, KIsomap cannot employ the characteristic of a kernel-based learning, *i.e*., a nonlinear kernel mapping, to explore higher order information of input data. Because the kernel idea of KIsomap is that the geodesic distance matrix with a constant-shifting technique is referred to be a Mercer kernel matrix [25].

To overcome the limitations of KIsomap mentioned above, in this paper a new kernel-based feature extraction method, called kernel discriminant Isomap (KDIsomap) is proposed. On one hand, KDIsomap considers both the intraclass scatter information and the interclass scatter information in a reproducing kernel Hilbert space (RKHS), and emphasizes the discriminant information. On the other hand, KDIsomap performs a nonlinear kernel mapping with a kernel function to extract the nonlinear features when mapping input data into some high-dimensional feature space. After extracting LBP features for facial representations, the proposed KDIsomap is used to produce the low-dimensional discriminant embedded data representations from the extracted LBP features with striking performance improvement on facial expression recognition tasks.

The remainder of this paper is organized as follows. The Local Binary Patterns (LBP) operator is described in Section 2. In Section 3, KIsomap is reviewed briefly and the proposed KDIsomap algorithm is presented in detail. In Section 4, two facial expression databases used for experiments are described. Section 5 shows the experiment results and analysis. Finally, the conclusions are given in Section 6.

## 2. Local Binary Patterns (LBP)

**Figure 1.**
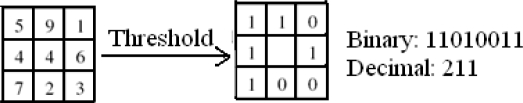
An example of basic LBP operator.

The original local binary patterns (LBP) [10] operator takes a local neighborhood around each pixel, thresholds the pixels of the neighborhood at the value of the central pixel and uses the resulting binary-valued image patch as a local image descriptor. It was originally defined for 3 × 3 neighborhoods, giving 8 bit codes based on the 8 pixels around the central one. The operator labels the pixels of an image by thresholding a 3 × 3 neighborhood of each pixel with the center value and considering the results as a binary number, and the 256-bin histogram of the LBP labels computed over a region is used as a texture descriptor. Figure 1 gives an example of the basic LBP operator.

**Figure 2.**
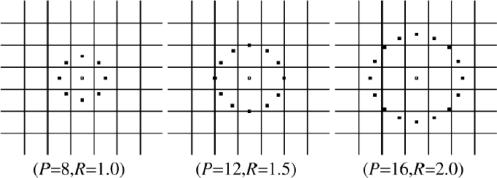
An example of the extended LBP with different (*P*, *R*).

The limitation of the basic LBP operator is that its small 3 × 3 neighborhood cannot capture the dominant features with large scale structures. As a result, to deal with the texture at different scales, the operator was later extended to use neighborhoods of different sizes. Figure 2 gives an example of the extended LBP operator, where the notation (*P*, *R*) denotes a neighborhood of *P* equally spaced sampling points on a circle of radius of *R* that form a circularly symmetric neighbor set. The second defined the so-called uniform patterns: an LBP is ‘uniform’ if it contains at most one 0-1 and one 1-0 transition when viewed as a circular bit string. For instance, 00000000, 001110000 and 11100001 are uniform patterns. It is observed that uniform patterns account for nearly 90% of all patterns in the (8, 1) neighborhood and for about 70% in the (16, 2) neighborhood in texture images. Accumulating the patterns which have more than 2 transitions into a single bin yields an LBP operator, ${\mathrm{LBR}}_{P,R}^{\mu 2}$, with less than 2*^P^* bins. Here, the superscript u2 in ${\mathrm{LBR}}_{P,R}^{\mu 2}$ indicates using only uniform patterns and labeling all remaining patterns with a single label.

After labeling an image with the LBP operator, a histogram of the labeled image *f_l_*(*x*, *y*) can be defined as:

(1) $$H_{i}=\sum_{x,y}I(f_{l}\;(x,y)=i),\;i=0,1,\cdots ,n-1$$

where *n* is the number of different labels produced by the LBP operator and:

(2) $$I(A)=\left\{ \begin{array}{l} 1,\;\text{A is true}\\ 0,\;\text{A is false} \end{array}\right.$$

This LBP histogram contains information about the distribution of the local micro-patterns, such as edges, spots and flat areas, over the whole image, so can be used to statistically describe image characteristics. For efficient face representation, face images were equally divided into *m* small regions *R*_1_, *R*_2_, ..., *R_m_* Once the *m* small regions *R*_1_, *R*_2_, ..., *R_m_* are determined, a histogram is computed independently within each of the *m* small regions. The resulting *m* histograms are concatenated into a single, spatially enhanced histogram which encodes both the appearance and the spatial relations of facial regions. In this spatially enhanced histogram, we effectively have a description of the face image on three different levels of locality: the labels for the histogram contain information about the patterns on a pixel-level, the labels are summed over a small region to produce information on a regional level and the regional histograms are concatenated to build a global description of the face image.

## 3. Our Method

In this section, we review the existing KIsomap algorithm in brief and explain the proposed KDIsomap algorithm in detail.

### 3.1. Review of KIsomap

The approximate geodesic distance matrix used in Isomap [20], can be interpreted as a Mercer kernel matrix. However, the kernel matrix based on the doubly centered geodesic distance matrix, is not always positive semi-definite. The method which incorporates a constant-shifting method into Isomap, is referred to as KIsomap [23], since the geodesic distance matrix with a constant-shifting technique is guaranteed to be a Mercer kernel matrix. This Mercer KIsomap algorithm has a good generalization property, enabling us to project new data points onto an associated low-dimensional manifold.

The KIsomap [23] operations can be summarized as follows:

Step 1: Identify the *k* nearest neighbors of each input data point and construct a neighborhood graph where edge lengths between points in a neighborhood are set as their Euclidean distances.
Step 2: Compute the geodesic distances, *d_ij_*, containing shortest paths for all pairs of data points by Dijkstra’ algorithm, and define $D^{2}=[d_{\mathrm{ij}}^{2}]$.
Step 3: Construct a matrix ***K***(***D***^2^) based on the approximate geodesic distance matrix:
  (3) $$K(D^{2})=-\frac{1}{2}{\mathrm{HD}}^{2}H$$
  where ***H*** = ***I*** – (1/*N*)*ee^T^*, e = [1,...,1]*^T^* ∈ *R^N^*.
Step 4: Compute the largest eigenvalue, c*, of the matrix $\left[\begin{array}{cc} 0 & 2K(D^{2})\\ -I & -4K(D) \end{array}\right]$ and construct a Mercer kernel matrix:
  (4) $$K^{*}=K(D^{2})+2cK(D)+\frac{1}{2}c^{2}H$$
  where K* is guaranteed to be positive semi-definite for *c* ≥ c^*^.
Step 5: Compute the top *d* eigenvectors of K*, which leads to the eigenvector matrix ***V***∈*R^N×d^* and the eigenvalue matrix *Λ^d×d^*.
Step 6: The embedded coordinates of the input points in the *d*-dimensional Euclidean space are given by:
  (5) $$Y=\Lambda^{\frac{1}{2}}V^{T}$$

### 3.2. The Proposed KDIsomap

The Fisher’s criterion in LDA [5], that is, the interclass scatter should be maximized while the intraclass scatter should be simultaneously minimized, has become one of the most important selection criteria for projection techniques since it endows the projected data vectors with a good discriminating power. Motivated by the Fisher’s criterion, when using KIsomap to extract the low-dimensional embedded data representations, the interclass dissimilarity could be maximized while the intraclass dissimilarity could be minimized in order to have improved tightness among similar patterns and better separability for dissimilar patterns. This can be realized by means of modifying the used Euclidean distance measure in the first step of KIsomap. In this section, we develop an improved kernelized variant of KIsomap by designing a kernel discriminant distance in a reproducing kernel Hilbert space (RKHS), which gives rise to the KDIsomap algorithm.

To develop the KDIsomap algorithm, a kernel matrix is firstly constructed by performing a nonlinear kernel mapping with a kernel function, and then a kernel discriminant distance, in which the interclass scatter is maximized while the intraclass scatter is simultaneously minimized, is designed to extract the discriminant information in a RKHS.

Given the input data point (*x_i_*, *L_i_*), where *x_i_* ∈ *R^D^* and *L_i_* is the class label of *x_i_*, the output data point is *y_i_* ∈ *R^d^* (*i* = 1, 2, 3, ..., *N*). The detailed steps of KDIsomap are presented as follows:

Step 1: Kernel mapping for each input data point *x_i_*.
  A nonlinear mapping function *φ* is defined as: *φ: R^D^* → ℱ, *x* ↦ *φ*(*x*)
  The input data point *x_i_* ∈ *R^D^* is mapping into an implicit high-dimensional feature space *F* with the nonlinear mapping function *φ*. In a RKHS, a kernel function *κ*(*x_i_*, *x_j_*) can be defined as:
  (6) $$\kappa (x_{i},x_{j})=\langle \varphi (x_{i}),\varphi (x_{j})\rangle =\varphi {\left(x_{i}\right)}^{T}\varphi (x_{j})$$
  where *κ* is known as a kernel.
Step 2: Find the nearest neighbors of each data point *φ*(*x_i_*) and construct a neighborhood graph where edge lengths between points in a neighborhood are set as the following kernel discriminant distance.
  The kernel Euclidean distance measure induced by a kernel *κ* can be defined as:
  (7) $$\begin{array}{lll} d_{\kappa }(x_{i},x_{j}) & = & \sqrt{\langle \varphi (x_{i})-\varphi (x_{j}),\varphi (x_{i})-\varphi (x_{j})\rangle }\\ & = & \sqrt{\kappa (x_{i},x_{i})-2\kappa (x_{i},x_{j})+\kappa (x_{j},x_{j})} \end{array}$$
  To preserve the intraclass neighbouring geometry, while maximizing the interclass scatter, a kernel discriminant distance in a RKHS is given as follows:
  (8) $$D_{\kappa }(x_{i},x_{j})=\{\begin{array}{ll} \sqrt{1-e^{\frac{-{\left[d_{\kappa }(x_{i},x_{j})\right]}^{2}}{\beta }}} & L_{i}=L_{j}\\ \sqrt{e^{\frac{{\left[d_{\kappa }(x_{i},x_{j})\right]}^{2}}{\beta }}}-\alpha & L_{i}\neq L_{j} \end{array}$$
  where *d_κ_*(*x_i_*, *x_j_*) is the kernel Euclidean distance matrix without class label information, whereas *D_κ_*(*x_i_*, *x_j_*) is the kernel discriminant distance matrix integrating class label information. β is a smoothing parameter related to the data ‘density’, and it is usually feasible to set β to be the average kernel Euclidean distance between all pairs of data points. α is a constant factor (0 ≤ α ≤ 1) and gives the intraclass dissimilarity a certain probability to exceed the interclass dissimilarity. As shown in Equation (8), we can make two observations. First, each dissimilarity function in *D_κ_*(*x_i_*, *x_j_*), *i.e.*, interclass dissimilarity and intraclass dissimilarity, is increasing monotonously with respect to the kernel Euclidean distance. This ensures that the main geometric structure of the original data sets can be preserved well when using KDIsomap to produce low-dimensional embedded data representations. Second, the interclass dissimilarity in *D_κ_*(*x_i_*, *x_j_*) can be always definitely larger than the intraclass dissimilarity, conferring a high discriminating power of KDIsomap’s projected data vectors. This is a good property for classification.
Step 3: Estimate the approximate geodesic distances.
Step 4: Construct a matrix ***K***(***D***^2^) based on ***K***(***D***^2^) = ***−*0.5*HD***^2^***H***.
Step 5: Compute the top *d* eigenvectors with the largest eigenvalue and give the embedded coordinates of the input points in the *d*-dimensional Euclidean space.

## 4. Facial Expression Database

Two popular facial expression databases, *i.e.*, the JAFFE database [9] and the Cohn-Kanade database [26], are used for facial expression recognition. Each database contains seven emotions: anger, joy, sadness, neutral, surprise, disgust and fear.

**Figure 3.**
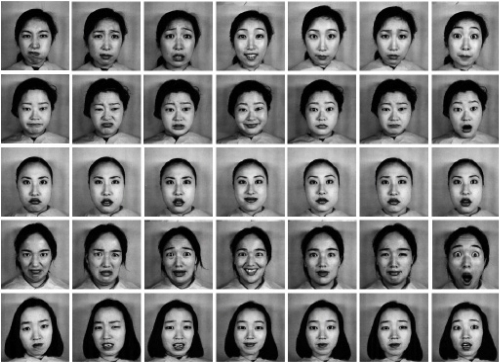
Examples of facial expression images from the JAFFE database.

The JAFFE database contains 213 images of female facial expressions. Each image has a resolution of 256 × 256 pixels. The head is almost in frontal pose. The number of images corresponding to each of the seven categories of expressions is roughly the same. A few of them are shown in Figure 3.

**Figure 4.**
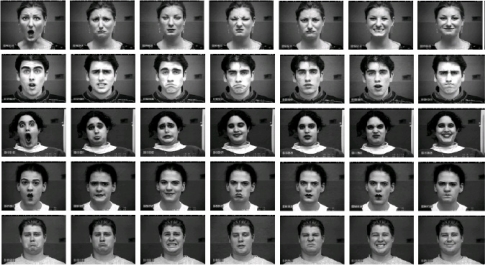
Examples of facial expression images from the Cohn-Kanade database.

The Cohn-Kanade database consists of 100 university students aged from 18 to 30 years, of which 65% were female, 15% were African-American and 3% were Asian or Latino. Subjects were instructed to perform a series of 23 facial displays, six of which were based on description of prototypic emotions. Image sequences from neutral to target display were digitized into 640 × 490 pixels with 8-bit precision for grayscale values. Figure 4 shows some sample images from the Cohn-Kanade database. As in [11,12], we selected 320 image sequences from the Cohn-Kanade database. The selected sequences, each of which could be labeled as one of the six basic emotions, come from 96 subjects, with 1 to 6 emotions per subject. For each sequence, the neutral face and three peak frames were used for prototypic expression recognition, resulting in 1,409 images (96 anger, 298 joy, 165 sadness, 225 surprise, 141 fear, 135 disgust and 349 neutral).

**Figure 5.**
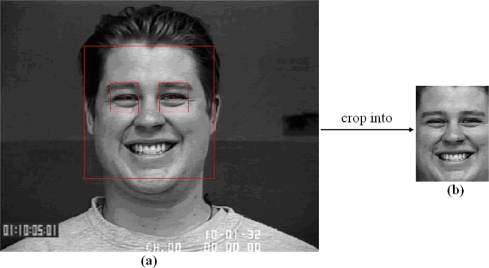
(**a**) Two eyes location of an original image from the Cohn-Kanade database. (**b**) The final cropped image of 110 × 150 pixels.

Following the setting in [11,12], we normalized the eye distance of face images to a fixed distance of 55 pixels once the centers of two eyes were located. Generally, it is observed that the width of a face is roughly two times the distance, and the height is roughly three times. Therefore, based on the normalized value of the eye distance, a resized image of 110 × 150 pixels was cropped from original image. To locate the centers of two eyes, automatic face registration was performed by using a robust real-time face detector based on a set of rectangle Harr-wavelet features [3]. From the results of automatic face detection including face location, face width and face height, two square bounding boxes for left eye and right eye were automatically constructed by using the geometry of a typical up-right face which has been widely utilized to find a proper spatial arrangement of facial features [27]. Then, the approximate center locations of two eyes can be automatically worked out in terms of the centers of two square bounding boxes for left eye and right eye. Figure 5 shows the detailed process of two eyes location and the final cropped image from the Cohn-Kanade database. No further alignment of facial features such as alignment of mouth was performed. Additionally, there was no attempt made to remove illumination changes due to LBP’s gray-scale invariance.

The cropped facial images of 110 × 150 pixels contain facial main components such as mouth, eyes, brows and noses. The LBP operator is applied to the whole region of the cropped facial images. For better uniform-LBP feature extraction, two parameters, *i.e*., the LBP operator and the number of regions divided, need to be optimized. Similar to the setting in [28], we selected the 59-bin operator ${\mathrm{LBR}}_{P,R}^{\mu 2}$, and divided the 110 × 150 pixels face images into 18 × 21 pixels regions, giving a good trade-off between recognition performance and feature vector length. Thus face images were divided into 42 (6 × 7) regions, and represented by the LBP histograms with the length of 2,478 (59 × 42).

## 5. Experimental Results and Analysis

To evaluate the performance of KDIsomap, facial expression recognition experiments were performed separately on the JAFFE database and the Cohn-Kanade Database. The performance of KDIsomap is compared with PCA [4], LDA [5], KPCA [24], kernel linear discriminant analysis (KLDA) [29], and KIsomap. The typical Gaussian kernel *κ*(*x_i_*, *x_j_*) = exp(−‖*x_i_* − *x_j_*‖^2^/2*σ*^2^) is adopted for KPCA, KLDA, KIsomap and KDIsomap, and the parameter *σ* is empirically set to 1 for its satisfying results. For simplicity the nearest neighbor classifier with the Euclidean metric is used for classification. Due to the computation complexity constraint, the embedded feature dimension is confined to the range [2,100] with an interval of 5. In each embedded dimension, the constant α (0 ≤ α ≤ 1) for KDIsomap can be optimized using a simple exhaustive search within a scope (α = 0, 0.1, 0.2, ..., 1). Note that the embedded dimensions of LDA and KLDA are limited to the range [2,6] because they can only find at most *c* − 1 meaningful embedded features, where *c* is the number of facial expression classes. Additionally, to detailedly explore the performance of all used methods in the low range [2,10], we present the recognition results of each embedded dimension with a small interval of 1.

A 10-fold cross validation scheme is employed for 7-class facial expression recognition experiments, and the average recognition results are reported. In detail, the data sets are split randomly into ten groups of roughly equal numbers of subjects. Nine groups are used as the training data to train a classifier, while the remaining group is used as the testing data. The above process is repeated ten times for each group in turn to be omitted from the training process. Finally, the average recognition results on the testing data are reported.

### 5.1. System Structure

**Figure 6.**
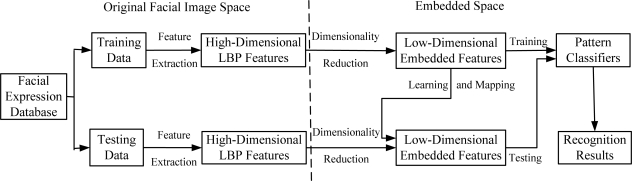
The basic system structure for facial expression recognition experiments using dimensionality reduction methods.

In order to clarify the experiment scheme of how to employ dimensionality reduction techniques such as PCA, LDA, KPCA, KLDA, KIsomap and KDIsomap on facial expression recognition tasks, Figure 6 presents the basic structure of a facial expression recognition system based on dimensionality reduction techniques.

It can be observed from Figure 6 that this system consists of three main components: feature extraction, feature dimensionality reduction and facial expression classification. In the feature extraction stage, the original facial images from the used facial expression databases are divided into two parts: training data and testing data. The corresponding LBP features for training data and testing data are extracted. The result of this stage is the extracted facial feature data represented by a set of high-dimensional LBP features. The second stage aims at reducing the size of LBP features and generating the new low-dimensional embedded features with dimensionality reduction techniques. It is noted that for the mapping of testing data, the low-dimensional embedded mapping of training data is needed to be learnt. This is realized by using the out-of-sample extensions of dimensionality reduction methods. For linear methods such as PCA and LDA, due to the linearity, their out-of-sample extensions are performed by multiplying testing data with the linear mapping matrix with a straightforward method. In other words, in PCA and LDA, the out-of-sample extension is computed by multiplying testing data with the linear mapping matrix obtained over training data. For nonlinear methods such as KPCA, KLDA, KIsomap and KDIsomap, their out-of-sample extensions are easily realized by a kernel trick as in KPCA [24]. The kernel trick first maps the original input data to another higher dimensional space with a kernel mapping function, and then performs the linear operations in this new feature space. The last stage in this system is in the low-dimensional embedded feature space the trained pattern classifiers like the nearest neighbor classifier are used to predict the accurate facial expression categories on testing data and the recognition results are given.

### 5.2. Experimental Results on the JAFFE Database

**Figure 7.**
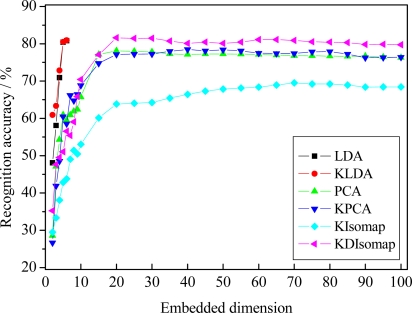
Performance comparisons of different methods on the JAFFE database.

The recognition results of different dimensionality reduction methods, *i.e.*, PCA, LDA, KPCA, KLDA, KIsomap and KDIsomap, are given in Figure 7. The best accuracy for different methods with corresponding embedded dimension is presented in Table 1. From the results in Figure 7 and Table 1, we can make four observations. Firstly, KDIsomap achieves the highest accuracy of 81.59% with 20 embedded features, outperforming the other methods, *i.e*., PCA, LDA, KPCA, KLDA and KIsomap. This shows that KDIsomap is able to extract the most discriminative low-dimensional embedded data representations for facial expression recognition. This can be attributed to the good property of KDIsomap for classification, *i.e.*, the interclass scatter is maximized while the intraclass scatter is simultaneously minimized. Secondly, KIsomap performs worst and obtains the lowest accuracy of 69.52%. The main reason is that KIsomap does not consider the class label information of data sets. In contrast, KDIsomap performs best. This reveals that KDIsomap makes an obvious improvement over KIsomap due to its supervised learning ability. Thirdly, two kernel methods, KPCA and KLDA, slightly outperform the corresponding non-kernel methods, *i.e*., PCA and LDA. This demonstrates the effectiveness of kernel methods. That is, they can employ the characteristic of a kernel-based learning to explore higher order information of input data. Finally, there is no significant improvement on facial expression recognition performance if more embedded feature dimensions are used. This shows that in our experiments it is acceptable that the embedded target dimension is confined to the range [2,100].

The recognition accuracy of 81.59% with basic LBP features and the nearest neighbor classifier is very encouraging, compared with the previously reported work [12] on the JAFFE database. In [12], using experimental settings similar to ours, the authors found the most discriminative LBP histograms using AdaBoost for better facial representation and then based on boosted-LBP features and SVM, they reported 7-class facial expression recognition accuracy of 79.8%, 79.8% and 81.0% for linear, polynomial and radial basis function (RBF) kernels, respectively. Nevertheless, in this study we did not used boosted-LBP features and SVM. To further compare the performance of KDIsomap with the work in [12], it’s an interesting task to explore the performance of boosted-LBP features and SVM integrating with KDIsomap in our future work.

To further explore the recognition accuracy per expression when KDIsomap performs best, Table 2 gives the confusion matrix of 7-class facial expression recognition results obtained by KDIsomap. From Table 2 we can see that two expressions, *i.e.*, anger and joy, are classified well with an accuracy of more than 90%, while other five expressions are discriminated with relatively low accuracy (less than 90%). In particular, sadness is recognized with the lowest accuracy of 61.88% since sadness is highly confused to neutral and fear.

### 5.3. Experimental Results on the Cohn-Kanade Database

**Figure 8.**
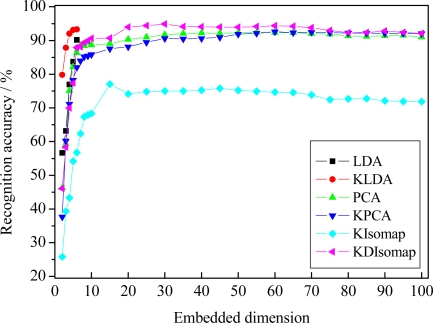
Performance comparisons of different methods on the Cohn-Kanade database.

Figure 8 presents the recognition performance of different dimensionality reduction methods on the Cohn-Kanade database. Table 3 shows the best accuracy for different methods with corresponding embedded dimension. The results in Figure 8 and Table 3 indicate that KDIsomap still obtains a recognition performance superior to that of other methods. In detail, among all used methods KDIsomap achieves the highest accuracy of 94.88% with 30 embedded features, whereas KIsomap gives the lowest accuracy of 75.81% with 40 embedded features. Again, this demonstrates the effectiveness of KDIsomap.

Table 4 gives the confusion matrix of 7-class expression recognition results when KDIsomap obtains the best performance. As shown in Table 4, we can see that 7-class facial expressions except sadness are identified very well with an accuracy of over 90%.

Compared with the previously reported work [11,12] with similar experimental settings to ours, the recognition performance of 94.88% is highly comparable. In [11], on 7-class facial expression recognition tasks they used LBP-based template matching to obtain an accuracy of 79.1%. Additionally, they also employed LBP-based SVM classifier to give an accuracy of 87.2%, 88.4% and 87.6% with linear, polynomial and RBF kernels, respectively. In [12], based on boosted-LBP features and SVM, on 7-class facial expression recognition tasks they reported an accuracy of 91.1%, 91.1% and 91.4% with linear, polynomial and RBF kernels, respectively.

### 5.4. Computational and Memory Complexity Comparison

The computational and memory complexity of a dimensionality reduction method is mainly determined by the target embedded feature dimensionality *d* and the number of training data points *n* (*d* < *n*). Table 5 presents a comparison of computational and memory complexity of different dimensionality reduction methods. The computational complexity demanding part of PCA and LDA is the eigenanalyis of a *d* × *d* matrix performed using a power method in *O*(*d*^3^). The corresponding memory requirement of PCA and LDA is *O*(*d*^2^). For the used kernel methods including KPCA, KLDA, KIsomap and KDIsomap, an eigenanalysis of an *n* × *n* matrix is performed using a power method in *O*(*n*^3^), so their computational complexity is *O*(*n*^3^). Since a full *n* × *n* kernel matrix is stored when performing KPCA, KLDA, KIsomap and KDIsomap, the memory complexity of these kernel methods is *O*(*n*^2^). As shown in Table 5, we can see that the proposed KDIsomap has the same computational and memory complexity as other kernel methods such as KPCA, KLDA and KIsomap.

## 6. Conclusions

In this paper, a new kernel-based manifold learning algorithm, called KDIsomap, is proposed for facial expression recognition. KDIsomap has two prominent characteristics. For one thing, as a kernel-based feature extraction method, KDIsomap can extract the nonlinear feature information embedded on a data set, as KPCA and KLDA do. For another, KDIsomap is designed to offer a high discriminating power for its low-dimensional embedded data representations in an effort to improve the performance on facial expression recognition. It’s worth pointing out that in our work we focus on facial expression recognition by using static images from two well-known facial expression databases, but we do not consider the temporal behaviors of facial expressions, which can potentially lead to more robust and accurate classification results. Therefore, it is also an interesting task to explore the performance of temporal information on facial expression recognition in our future work.

## Figures and Tables

**Table 1.** The best accuracy (%) of different methods on the JAFFE database.

**Method**	**PCA**	**LDA**	**KPCA**	**KLDA**	**KIsomap**	**KDIsomap**
Dimension	20	6	40	6	70	20
Accuracy	78.09 ± 4.2	80.81 ± 3.6	78.47 ± 4.0	80.93 ± 3.9	69.52 ± 4.7	81.59 ± 3.5

**Table 2.** Confusion matrix of 7-class facial expression recognition results obtained by KDIsomap on the JAFFE database.

	
	**Anger (%)**	**Joy (%)**	**Sadness (%)**	**Surprise (%)**	**Disgust (%)**	**Fear (%)**	**Neutral (%)**
**Anger**	**90.10**	0	3.58	0	3.32	0	3.00
**Joy**	0	**93.54**	3.12	0	0	0	3.34
**Sadness**	6.45	3.21	**61.88**	0	3.29	9.68	15.49
**Surprise**	0	3.13	3.54	**86.67**	0	6.66	0
**Disgust**	7.42	0	3.68	0	**81.48**	7.42	0
**Fear**	0	0	12.48	6.25	3.13	**78.14**	0
**Neutral**	0	0	17.23	3.45	0	0	**79.32**

**Table 3.** The best accuracy (%) of different methods on the Cohn-Kanade database.

**Method**	**PCA**	**LDA**	**KPCA**	**KLDA**	**KIsomap**	**KDIsomap**
Dimension	55	6	60	6	40	30
Accuracy	92.43 ± 3.3	90.18 ± 3.0	92.59 ± 3.6	93.32 ± 3.0	75.81 ± 4.2	94.88 ± 3.1

**Table 4.** Confusion matrix of 7-class facial expression recognition results obtained by KDIsomap on the Cohn-Kanade database.

	
	**Anger (%)**	**Joy (%)**	**Sadness (%)**	**Surprise (%)**	**Disgust (%)**	**Fear (%)**	**Neutral (%)**
**Anger**	**97.60**	0	0.96	0	0	1.44	0
**Joy**	0.31	**95.53**	0.28	0	1.97	0.30	1.61
**Sadness**	2.15	1.02	**89.84**	0	5.76	0	1.23
**Surprise**	0.24	0.24	1.99	**97.18**	0	0	0.35
**Disgust**	0	1.16	1.28	3.00	**94.21**	0.35	0
**Fear**	0	0	0	0.38	0	**99.62**	0
**Neutral**	2.12	1.79	3.27	0.44	1.79	0.44	**90.15**

**Table 5.** Computational and memory complexity of different dimensionality reduction methods.

**Method**	**PCA**	**LDA**	**KPCA**	**KLDA**	**KIsomap**	**KDIsomap**
Computational complexity	*O*(*d*^3^)	*O*(*d*^3^)	*O*(*n*^3^)	*O*(*n*^3^)	*O*(*n*^3^)	*O*(*n*^3^)
Memory complexity	*O*(*d*^2^)	*O*(*d*^2^)	*O*(*n*^2^)	*O*(*n*^2^)	*O*(*n*^2^)	*O*(*n*^2^)
